# Incidence of deep vein thrombosis and symptomatic pulmonary embolism in Taiwanese patients with pelvic and/or acetabular fractures: a retrospective study

**DOI:** 10.1038/s41598-023-43449-4

**Published:** 2023-09-28

**Authors:** Po-Meng Hsiao, Shu-Chen Liao, I.-Jung Chen, Ying-Chao Chou, Yung-Heng Hsu, Shu-Mei Wang, Yi-Hsun Yu

**Affiliations:** 1 Department of Orthopaedics, New Taipei Municipal TuCheng Hospital, No. 6, Sec. 2, Jincheng Rd., Tucheng Dist., New Taipei City, 236 Taiwan; 2 Chang Gung University, No. 259, Wenhua 1St Rd., Guishan Dist., Taoyuan City, 33302 Taiwan; 3 Department of Orthopedic Surgery, Musculoskeletal Research Center, Chang Gung Memorial Hospital, Linkou Branch, No. 5, Fu-Hsin St. Kweishan, 33302 Taoyuan, Taiwan; 4 Department of Pediatric Orthopedics, Chang Gung Memorial Hospital, No. 5, Fu-Hsin St. Kweishan, 33302 Taoyuan, Taiwan

**Keywords:** Risk factors, Epidemiology, Trauma, Thrombosis

## Abstract

Venous thromboembolism (VTE) is common in patients with trauma, and thromboprophylaxis has been advocated. However, conflicting results regarding VTE rates in the Asian population following orthopaedic procedures have been presented. We aimed to investigate the VTE incidence in Taiwanese patients with pelvic and/or acetabular fractures and identify the associated risk factors. We included 402 patients who underwent surgery for pelvic and/or acetabular fractures. All patients received mechanical thromboprophylaxis with graduated compression stockings. Duplex scanning was performed postoperatively or during follow-up when signs or symptoms of deep vein thrombosis (DVT) developed. Variables with a significance level of ≤ 0.1 in the univariate analyses were introduced into the multivariate logistic regression analysis to identify DVT risk factors. The overall DVT and symptomatic pulmonary embolism (PE) rate was 3.48% (14/402 patients). Among patients with DVT, 46.1% were asymptomatic. Patients with VTE were significantly older than those without. Multivariate logistic regression analysis revealed that age was a VTE risk factor. The incidence of DVT and symptomatic PE in our cohort was low. Advanced age was a risk factor for VTE. These findings could help clinicians develop appropriate prevention and treatment strategies for VTE in Taiwanese patients with pelvic and/or acetabular fractures.

## Introduction

The reported incidence of venous thromboembolism (VTE), including deep vein thrombosis (DVT) and pulmonary embolism (PE) in patients with pelvic and/or acetabular fractures, is as high as 27–41%^1–6^. The use of pharmacological and mechanical thromboprophylaxis for VTE in these patients is strongly recommended in the existing literature and widely accepted in current clinical practice^7–9^. However, pharmacological thromboprophylaxis is associated with side effects, including the risk of increased bleeding, hematoma formation, persistent wound drainage, and infection, and the need for red blood cell transfusion^10–13^. Further, some medications used for pharmacological thromboprophylaxis require close monitoring, while others have no antidotes for overdosage, which poses an additional treatment challenge.

Pelvic and acetabular fractures usually result from high-energy trauma. Associated injuries, such as vascular injuries, multiple fractures, visceral organ lacerations, and intracranial haemorrhages, are not uncommon^14^. In such situations, bleeding control during treatment is a key concern, and pharmacological thromboprophylaxis is not recommended^8^. Over years of practice in our institute, pharmacological thromboprophylaxis was not routinely used for patients with pelvic and/or acetabular trauma; despite this, symptomatic VTE rarely occurs. In fact, there is a growing body of literature describing the rarity of VTE in Asian populations, even without the use of pharmacological or mechanical thromboprophylaxis perioperatively^15–19^. However, most of these studies have focused on elective and non-trauma surgeries, such as arthroplasty. Several studies have claimed that the incidence of VTE in Asian populations is similar to that of Western populations, especially in those with pelvic and/or acetabular fractures^1,3–6,20,21^, which is contrary to our observations over time. Therefore, we aimed to investigate the incidence of DVT and symptomatic PE in Taiwanese patients with pelvic and/or acetabular fractures without pharmacological thromboprophylaxis and associated risk factors.

## Methods

After obtaining approval from the institutional review board, 439 patients with pelvic and/or acetabular fractures treated surgically at our level I trauma center between March 2016 and March 2020 were identified and their data were retrospectively reviewed. The study was conducted in accordance with the guidelines of the Declaration of Helsinki and approved by the Ethics Committee of the Chang Gung Medical Foundation (protocol code 202100049B0, approved on 2021/01/20). The review board waived the requirement for informed consent due to the retrospective nature of the study.

The inclusion criteria were Taiwanese patients with pelvic and/or acetabular fractures treated with any surgical method. Patients with conditions predisposing them to VTE, including a history of cancer, concurrent use of hormone therapy, history of venous thromboembolism, presence of varicose veins in the lower extremities, and regular anticoagulant use, were excluded. We excluded 17 (3.9%) patients with missing data (11 patients did not undergo lower-limb duplex ultrasound evaluation of the peripheral vessels due to mortality, concomitant bilateral lower-limb injury, or other unspecified reasons, and six patients with no evidence of DVT had incomplete laboratory data or relevant medical records). Twenty (4.6%) patients were classified as having fragility fractures of the pelvis according to the low-energy trauma mechanism and fracture patterns and were excluded from the study group, leaving 402 patients for the final analysis.

### Clinical management and evaluation

On the first day of admission, we applied bilateral thigh-length graduated compression stockings to all patients without contraindications such as lower-leg fracture or soft tissue injuries. Patients with unilateral lower-limb injury were instructed to use stockings on the uninjured limb. To reduce the immobilisation time, definite fixation of the pelvic and/or acetabular fracture was performed as soon as the patient was sufficiently stable. Duplex scanning of the bilateral lower limbs or the leg with intact soft tissue was performed by a cardiologist 3 days postoperatively. Thigh-length graduated compression stockings were used for 12 weeks after the operation.

Patients were assisted to sit at the bedside the day after surgery, if tolerable. Rehabilitation consisted of stretching and strengthening of the thigh and calf muscles, as instructed by a physiotherapist. Ambulation with a walker with partial weight bearing on the injured side was encouraged. Patients with bilateral involvement were encouraged to move in a wheelchair to prevent prolonged bed rest. Clinical follow-up was conducted at 2 weeks, 6 weeks, 12 weeks, 6 months, and 1 year postoperatively. Patients with negative findings for DVT on sonography were educated regarding the signs and symptoms of DVT, including swelling, redness, local heat, pain, and tenderness in the lower limb, particularly calf pain on foot dorsiflexion (Homan’s sign), and were asked to return to the hospital in case of any doubt. All patients were instructed to return to the emergency room if they developed signs or symptoms of PE, including shortness of breath and chest discomfort. The patient’s age, sex, body mass index (BMI), injury severity score, associated fractures, time to surgery, perioperative value of the international normalised ratio, preoperative and postoperative serum d-dimer levels, estimated amount of intraoperative blood loss, units of packed red blood cells (PRBC) and fresh frozen plasma (FFP) transfused, and operation duration were recorded. Patients were then grouped into VTE and non-VTE groups for analysis.

### Statistical analysis

Numerical variables were tested using the Shapiro–Wilk test for normality. Data are presented as median (25th quartile, 75th quartile, interquartile range [IQR]). Describable data are presented as numbers and percentages. The Mann–Whitney U test was used for comparisons between continuous variables, and Fisher’s exact or Chi-square test was used for categorical data. Variables with a significance level of 0.1 or below, such as age, BMI, estimated amount of intraoperative blood loss, units of PRBC and FFP transfused, and serum d-dimer level > 10,000 ng/ml before and after the operation, were considered potential risk factors and introduced into the multivariate logistic regression analysis. Variables with significance at 0.05 level in the multivariate analysis were regarded as independent risk factors. All statistical analyses were performed using Statistical Package for the Social Sciences (version 26.0, IBM Corp. Armonk, NY).

## Results

VTE was identified in 14 out of 402 (3.48%) patients. One (0.25%) patient had a PE, 13 (3.23%) had lower limb DVTs, and six (46.2%) patients with DVT were asymptomatic. All positive findings of VTE were identified on postoperative day 3 by duplex scanning or computed tomography angiography. No patients developed significant symptoms or signs of VTE during the follow-up. The details of the patients with VTE are listed in Table 1, and their clinical characteristics are shown in Table 2. The patient who developed symptomatic PE had a pelvic fracture, anteroposterior compression type III, and a right femur subtrochanteric fracture. The patient experienced sudden desaturation at the end of the surgery for both fractures. The surgery was halted, and the patient underwent extracorporeal membrane oxygenation (ECMO) implantation followed by pulmonary embolectomy under cardiopulmonary bypass the next day. The patient survived, and his clinical course was complicated by the development of toe gangrene from ECMO.

**Table 1:** Details of patients with venous thromboembolism.

Patient	Sex/age (years)	VTE	AO fracture classification	Approach	Treatment	Time to surgery (days)	Surgery duration (minutes)
1	M/49	DVT	61-B2 + 62-B3	AIP + 1st window	ORIF + PCS	6	226
2	F/69	DVT	61-B2 + 61-B1	KL	ORIF	13	133
3	F/48	DVT	61-C1	IL	ORIF	4	166
4	M/42	DVT	61-C1	MIS	ISS + ACS	4	99
5	F/75	DVT	61-B3	AIP + MIS	ORIF + TITS	8	144
6	F/54	DVT	61-B3	AIP + MIS	ORIF + TITS	13	200
7	F/75	DVT	61-B2	AIP	ORIF	25	349
8	M/31	DVT	62-B1	MIS	ACS	34	237
9	F/62	DVT	62-C2	Pararectus	ORIF	9	192
10	M/72	DVT	62-A1	Modified Gibson	ORIF	18	220
11	M/54	DVT	62-C2	Extended IL	ORIF	29	277
12	F/44	DVT	61-C1	Posterior	SPO	16	216
13	F/43	DVT	62-C3	AIP + 1st window	ORIF	9	210
14	M/67	PE	61-B3	AIP	ORIF	29	149

AO, Arbeitsgemeinschaft für Osteosynthesefragen; VTE, venous thromboembolic event; DVT, deep vein thrombosis; PE, pulmonary embolism; AIP, anterior intra-pelvic approach; IL, ilioinguinal approach; MIS, minimal invasive surgery; ORIF, open reduction and internal fixation; SPO, spinopelvic fixation; ISS, iliosacral screw; TITS, transiliac-transsacral screw; ACS, anterior column screw; PCS, posterior column screw.

**Table 2:** Demographics, clinical characteristics.

Variables	VTE group	Non-VTE group	Odds ratio	95% CI	p-value
Number of patients	14 (3.5%)	388 (96.5%)			
Male	6 (1.5%)	227 (56.5%)	0.530	0.181–1.563	1.358
Female	8 (1.0%)	161 (40%)	1.880	0.640–5.522	1.358
Age (years)	54.3 (45.1–68.4)	34.4 (23.0–52.5)	1.065	1.029–1.103	< 0.001
BMI (kg/cm^2^)	28.4 (26.0–29.4)	23.5 (21.3–27.0)	1.129	1.029–1.24	0.001
ISS	13 (8.3–17.5)	9 (9–20)	1.012	0.965–1.061	0.587
Time to surgery (days)	8 (6–10)	7 (5–9)	1.046	0.923–1.185	0.300
Surgery duration (min)	205 (153–219)	168 (129–235)	1.002	0.996–1.008	0.267
Blood loss (ml)	600 (425–737)	300 (100–700)	1.000	0.999–1.001	0.071
Patients with multiple fractures	7 (17.4%)	209 (52.6%)	0.856	0.295–2.295	0.776
Blood transfused (unit)					
PRBC	6 (4.0–11.5)	4 (4.0–10.0)	1.036	0.987–1.087	0.089
FFP	2 (2.0–7.0)	2 (2.0–6.0)	1.02	0.942–1.104	0.098
Pelvic fracture	6 (1.5%)	212 (52.7%)	0.623	0.212–1.828	0.385
Acetabular fracture	4 (1.0%)	121 (30.1%)	0.883	0.271–2.870	1.000
Both fracture	4 (1.0%)	55 (13.7%)	2.422	0.734–7.993	0.134
d-dimer (ng/ml)					
Pre-op > 10,000	10 (2.5%)	152 (37.8%)	3.882	1.196–12.598	0.016
Post-op > 10,000	9 (2.2%)	126 (31.3%)	3.743	1.229–11.399	0.013

Continuous variables are presented as median (IQR) and analysed using the Mann–Whitney U test for p-values and using the univariate logistic regression test for odds ratios and 95% CIs. Categorical variables are presented as number (percentage of 402 patients) and analysed with Fisher’s exact or Chi-square test for p-value, odds ratios, and 95% CIs. BMI, body mass index; ISS, injury severity score; PRBC, packed red blood cell; FFP, fresh frozen plasma; CI, confidence interval.

In the univariate analyses, variables such as age, BMI, estimated amount of intraoperative blood loss, units of PRBC and FFP transfused, and perioperative serum d-dimer level > 10,000 ng/ml had p-values ≤ 0.1. These potential factors were introduced into the multivariate logistic regression analysis, and age (odds ratio 1.060 [95% confidence interval (CI) 1.024–1.099]; p = 0.001) was the only significant variable. Patient age was significantly higher in the VTE group (median: 54.3 years [IQR: 45.1–68.4]) than in the non-VTE group (median: 34.4 years [IQR: 23.0–52.5]).

## Discussion

The overall rates of DVT and symptomatic PE were 3.48%, all of which developed within postoperative day 3. Among all patients with DVT, 46.1% were asymptomatic. Patients with VTE were significantly older than those without VTE. Multivariate logistic regression analysis revealed that age was a risk factor for VTE.

While pharmacological thromboprophylaxis effectively reduces the incidence of VTE^20,21^, it also has some limitations and side effects such as increased bleeding risk, haematoma formation, wound complications, and the need for red blood cell transfusion^10–13^. In our institution, pharmacological thromboprophylaxis has not been performed in daily clinical practice over the years; despite this, VTE rarely occurs, which is contrary to the current literature^1,3–6,20,21^. Therefore, we screened all patients who underwent surgery for traumatic pelvic and/or acetabular fractures with peripheral duplex scanning since March 2016 to avoid missed diagnoses of asymptomatic DVT. However, the results were mostly negative for DVT. The medical records of the patients were retrospectively reviewed to evaluate the incidence of VTE.

The incidence of VTE in patients with pelvic and/or acetabular fractures is reported to be as high as 41%^1–6^. Lowe et al. reviewed their database in the United States and reported unpreventable VTE in nine of 510 (1.7%) pelvic fractures and one of 210 (0.42%) acetabular fractures, even with strict adherence to modern prophylactic guidelines^20^. However, some studies have reported a large proportion of asymptomatic VTE in patients with pelvic and/or acetabular fractures^4,22^. In our study, 42.9% (6/14) of the patients with VTE showed no signs or symptoms. As only patients with symptomatic VTE confirmed by non-invasive imaging were included in the study by Lowe et al., the actual incidence of unpreventable VTE in the US population might be underestimated.

Asians are less susceptible to VTE, as evidenced in several studies of patients who underwent elective arthroplasty surgery^15–19,23,24^. Kang et al. found an overall low incidence of VTE (0.8%, 8/992 patients) in Asian patients who underwent total hip replacement (THR)^16^. Lee et al. published a meta-analysis on the rates of DVT and symptomatic PE in Asian patients after total knee replacement (TKR) and concluded that the incidence was low (0.01% for symptomatic PE; 1.9% for symptomatic DVT)^19^. One possible explanation for this may be the genetic differences. Factor V Leiden mutations increase the risk of thromboembolism; in particular, homozygosity carries a 50- to 100-fold higher risk. This mutation is found in 4–6% of the US population but is rare in Asian populations^25^. The prothrombin G20210A mutation is most commonly seen in Europeans and is less prevalent in Asians, and patients with the 20210A allele have a relative risk of thrombosis of 2.8 (95% CI: 1.4 to 5.6)^26^. Kim et al. and Kim et al. found an absence of the Factor V Leiden and G20210A mutations in all 1063 patients receiving THR and TKR^17,18^.

Although Asians are genetically protected against VTE, several studies have reported similar VTE rates in both Asian and Western populations with pelvic and/or acetabular fractures (Table 3)^1,3–6^. One explanation for the high rates of VTE in these studies was prolonged immobilisation. Wang et al. reported an overall VTE rate of 29.09% in patients with pelvic and acetabular fractures, but in 33 out of 110 (30%) patients, the time to surgery was over 2 weeks, and 55% (18/33) of these patients were associated with VTE^6^. Kim et al. reported that the VTE rate in Korean patients with pelvic and/or acetabular fractures was 33.7% (32/95)^1^. In their study, 81 (85.3%) patients underwent surgery at a median of 26 days (IQR: 0–42), and 26 (32.1%) of these patients developed VTE. Fourteen patients did not undergo surgery, and six (42.9%) developed VTE. Niikura et al. documented 19 VTE cases in 46 (41.4%) Japanese patients with pelvic or acetabular fractures^4^. In their study, four (21%) patients who developed VTE received only conservative treatment, and five (26%) received external fixation. These results suggest that these therapeutic strategies may not be adequate to allow patients to start early mobilisation and thus predispose them to VTE. However, contrary to these studies, only 8.5% (34/402) of our patients had a time to surgery of > 14 days, and 54% of our patients underwent surgery within 7 days. Therefore, we assumed that early surgical intervention and mobilisation in our patients may account for the lower VTE rate. However, further studies are required to investigate the factors that account for this discrepancy.

**Table 3:** Published studies of the incidence of VTE in pelvic or acetabular fractures.

Study	Patient number	VTE number	Overall incidence (%)	Prophylaxis	Screening method	Risk factors
Montgomery et al.^2^	101	38	38	P + IVCF	MRV	Time to surgery
Stannard et al.^27^	107	15	14	M	U + MRV	Age, time to surgery
Steele et al.^21^	103	11	11	P	U	High ISS, delayed use of LMWH
*Kim et al.^11^	95	32	34	N/A	CTV	Age, VS type fracture
*Sen et al.^5^	56	15	26.8	None	CTV	None
*Wang et al.^6^	95	32	34	N/A		Age, time to surgery
*Niikura et al.^3^	126	38	30	M	U, CT (+)	Multiple fractures
*Niikura et al.^4^	46	19	41	M	U, CT (+)	N/A
Lowe et al.^20^	750	10	1	M + P	N/A	None

VTE, venous thromboembolic event; P, pharmacological thromboprophylaxis; IVCF, inferior vena cava filter; M, mechanical thromboprophylaxis; MRV, magnetic resonance venography; U, ultrasonography; CTV, computed tomography venography; CT (+), computed tomography with contrast; ISS, injury severity score; LMWH, low molecular weight heparin; VS, vertical shear; N/A, not applicable.

*Studies reporting the incidence of VTE in Asian patients with pelvic and/or acetabular fracture.

Various risk factors for VTE in patients with pelvic or acetabular fractures have been described (Table 3)^1–6,20,21,27^. After multivariate logistic regression analysis, our results were consistent with those of other studies, which showed that advanced age was a risk factor for VTE (median: 54.3 years for the VTE group [IQR: 45.1–68.4] versus 34.4 years for the non-VTE group [IQR: 23.0–52.5]; p = 0.001). Studies have shown that the risk of thrombosis increases significantly with age^28–30^. The cause of this strong correlation has not yet been thoroughly investigated. Some postulated factors include endothelial dysfunction, reduced mobility, comorbidities, and increased levels of coagulation factors^28–30^. More stringent adherence to thromboprophylaxis guidelines may be needed in older populations.

In our study, 40.3% of all patients had serum d-dimer levels of > 10,000 ng/mL at the time of surgery. In univariate analysis, a perioperative serum d-dimer level > 10,000 ng/mL was associated with an increased risk of VTE. Nevertheless, after multivariate logistic regression analysis, VTE was not correlated with d-dimer levels above or below 10,000 ng/ml. Similar to our findings, Wang et al. defined a serum d-dimer level > 1.4 mg/L (14,000 ng/ml) as positive in their study and found limited significance of serum d-dimer levels to predict DVT (p = 1.000)^6^. The use of d-dimer levels has long been criticised for its poor specificity in detecting or excluding VTE^31–33^. Abnormal serum d-dimer levels could be present in patients without thrombosis^31^, whereas thrombosis may be present in patients with normal serum d-dimer levels^32,33^. Niikura et al. suggested monitoring the dynamic status of the serum d-dimer level rather than its value at a single time point^4^. They recommended paying special attention to patients whose serum d-dimer levels remained higher than 20 μg/ml (20,000 ng/ml) 5 days after injury or surgery. Therefore, serum d-dimer levels should be interpreted with caution in trauma patients. Unnecessary overuse of pharmacological thromboprophylaxis, advanced screening tools, or delays in surgery should be avoided if no symptoms or signs of VTE are noted.

This study had some limitations. First, patients with severe bilateral lower-limb soft tissue injuries were excluded because duplex ultrasonography could not be performed in such cases. This could have created a selection bias, as patients with profound lower-limb soft tissue damage are theoretically more susceptible to DVT from damaged vessel walls and prolonged immobilisation^29^. Excluding these patients may have underestimated the overall VTE rate. However, these patients did not receive pharmacological thromboprophylaxis and showed no signs or symptoms of VTE after resolution of soft tissue problems. Therefore, we assumed that their exclusion would have little effect on the incidence of VTE in our study. Second, duplex scanning was performed 3 days postoperatively or whenever signs of DVT were noted during the follow-up period. Therefore, we may have missed some patients with late-onset asymptomatic DVT. However, late-onset VTE after pelvic surgery has been previously reported and is limited to 0.83% within 90 days of surgery^34^; none of our patients developed signs or symptoms of VTE during clinical follow-up. Therefore, we assume that the overall rate would not change significantly. Third, owing to the limitations of the laboratory at our institute, we were unable to obtain precise values of serum d-dimer levels > 10,000 ng/mL; rather, these were labelled as “ > 10,000.” Therefore, we could not obtain continuous data for statistical purposes, and a cut-off value could not be calculated to evaluate the usefulness of predicting VTE with the serum d-dimer level. However, a serum d-dimer level above or below 10,000 ng/mL was not a significant variable in the multivariate logistic regression analysis. Additionally, the limitations of d-dimer levels to predict VTE have been mentioned in the literature^31–33^.

## Conclusion

The incidence of DVT and asymptomatic PE in Taiwanese patients with pelvic and/or acetabular fractures was low in the present study. However, special attention should be paid to the older population, as age is an independent risk factor for DVT. These results do not suggest that pharmacological thromboprophylaxis should be withheld for all Taiwanese patients. However, these findings could help clinicians develop targeted treatment strategies to prevent VTE and bleeding complications associated with thromboprophylaxis. Future studies are warranted to further elucidate the reason for the difference in the VTE incidence among Asians with pelvic and/or acetabular fractures.

## Data Availability

The datasets used and/or analysed during the current study are available from the corresponding author upon reasonable request.
